# A Novel Multiplex Real-Time PCR for the Identification of Mycobacteria Associated with Zoonotic Tuberculosis

**DOI:** 10.1371/journal.pone.0023481

**Published:** 2011-08-09

**Authors:** Kate Reddington, Justin O'Grady, Siobhan Dorai-Raj, Stefan Niemann, Dick van Soolingen, Thomas Barry

**Affiliations:** 1 Microbiology, School of Natural Sciences, National University of Ireland, Galway, Ireland; 2 Molecular Diagnostics Research Group, National Centre for Biomedical Engineering Science (NCBES), National University of Ireland, Galway, Ireland; 3 Molecular Mycobacteriology, Research Center Borstel, Borstel, Germany; 4 National Tuberculosis Reference Laboratory, National Institute for Public Health and the Environment, Bilthoven, The Netherlands; Queen Mary University of London, United Kingdom

## Abstract

**Background:**

Tuberculosis (TB) is the leading cause of death worldwide from a single infectious agent. An ability to detect the *Mycobacterium tuberculosis* complex (MTC) in clinical material while simultaneously differentiating its members is considered important. This allows for the gathering of epidemiological information pertaining to the prevalence, transmission and geographical distribution of the MTC, including those MTC members associated with zoonotic TB infection in humans. Also differentiating between members of the MTC provides the clinician with inherent MTC specific drug susceptibility profiles to guide appropriate chemotherapy.

**Methodology/Principal Findings:**

The aim of this study was to develop a multiplex real-time PCR assay using novel molecular targets to identify and differentiate between the phylogenetically closely related *M. bovis*, *M. bovis* BCG and *M. caprae*. The *lpqT* gene was explored for the collective identification of *M. bovis*, *M. bovis* BCG and *M. caprae*, the *lepA* gene was targeted for the specific identification of *M. caprae* and a Region of Difference 1 (RD1) assay was incorporated in the test to differentiate *M. bovis* BCG. The multiplex real-time PCR assay was evaluated on 133 bacterial strains and was determined to be 100% specific for the members of the MTC targeted.

**Conclusions/Significance:**

The multiplex real-time PCR assay developed in this study is the first assay described for the identification and simultaneous differentiation of *M. bovis*, *M. bovis* BCG and *M. caprae* in one internally controlled reaction. Future validation of this multiplex assay should demonstrate its potential in the rapid and accurate diagnosis of TB caused by these three mycobacteria. Furthermore, the developed assay may be used in conjunction with a recently described multiplex real-time PCR assay for identification of the MTC and simultaneous differentiation of *M. tuberculosis*, *M. canettii* resulting in an ability to differentiate five of the eight members of the MTC.

## Introduction

Tuberculosis (TB) in humans is mostly caused by members of the *Mycobacterium tuberculosis* complex (MTC), although infections caused by nontuberculous mycobacteria (NTM) can mimic this disease [1]. *M. tuberculosis* is considered to be the primary pathogen of TB in humans [2]. Other members of the MTC associated with human TB infection include *M. canettii* and *M. africanum,* with the highest prevalence in various parts of Africa [3]. The remaining members of the MTC including *M. bovis*, *M. caprae*, *M. microti* and *M. pinnipedii* are considered predominantly animal pathogens, apart from the, for vaccination purposes, attenuated *M. bovis* BCG strain. However, all members of the MTC have been shown to cause human TB infection [4]. For example, the major etiological agents of zoonotic TB in humans are the phylogenetically related *M. bovis* and *M. caprae* [5]. These members of the MTC occur worldwide and there are indications which suggest the true prevalence of zoonotic human TB infection may be underrepresented, especially because of the extrapulmonary manifestation of the disease generally caused by these bacteria [6], [7], [8]. In developed countries it has been suggested that the burden of bovine TB in humans ranges from 0.5 to 7.2% of TB cases, while in developing countries, where very little data is available, this figure may be as high as 15% [9], [10].

The diagnosis of TB in humans in high prevalence setting usually still relies on smear microscopy, although this is insensitive, especially in patients co-infected with HIV [11], [12].The gold standard culture method is both time consuming and laborious [13]. Therefore, *in vitro* nucleic acid amplification tests (NAATs) offer a rapid and sensitive alternative to traditional methods for the detection of TB infection, with an increasing number of commercially available TB diagnostics kits becoming available [14], [15], [16], in addition to NAAT methods described in the literature [17], [18]. However, most of these rapid and traditional diagnostics methods detect the presence of the MTC but do not identify the specific etiological agent of infection.

Since the introduction of pasteurisation and bovine TB eradication programmes in developed countries there has been a significant reduction in the number of zoonotic TB cases in humans [10], with the majority of such cases occurring as a result of endogenous reactivation of a previous infection [9], [19]. However, recent reports have identified TB in humans caused by *M. bovis* in countries officially free from bovine TB [8] and suggest that the true prevalence of zoonotic TB may be underestimated clinically [7]. In addition, recent reports show that *M. bovis* infection in younger patient groups in the Western world are still common, most likely owing to consumption of raw milk (products) in the country of origin [20]. Moreover, zoonotic TB remains a significant threat to human health in developing countries where its prevalence is currently unknown, as differentiation between the members of the MTC is not routinely performed [9], [21].

The accurate differentiation of the MTC is also clinically important, due to varying natural resistances of members of the MTC to anti-TB drugs. [22]. As such, the ability to differentiate between members of the MTC could potentially allow for the monitoring of zoonotic exposure leading to human TB infection, in addition to providing accurate epidemiological information for the clinician, guiding contact tracing and source case finding.

In this study, we report the design, development and testing of a multiplex real-time PCR assay, using novel nucleic acid molecular targets, to identify and simultaneously differentiate between *M. bovis*, *M. bovis* BCG and *M. caprae* in one internally controlled reaction. This assay can be used independently, or in combination with, a recently described multiplex PCR assay for the identification of the MTC, *M. tuberculosis* and *M. canettii* [23].

## Materials and Methods

### Diagnostics target identification

The diagnostics target genes used in this study were identified using a number of experimental and bioinformatics approaches. Potential target regions were identified using the Mycobacterial Genome Divergence Database (MGDD) (http://mirna.jnu.ac.in/mgdd/), which allowed for identification of insertions, deletions and single nucleotide polymorphisms between *M. tuberculosis*, *M. bovis* and *M. bovis* BCG. Other potential target regions were identified using the web based version of the Artemis comparison tool, WebACT (http://www.webact.org/WebACT/home). Nucleotide sequence information for *M. africanum*, *M. microti* and *M. canettii* was retrieved from the Welcome Trust Sanger Institute website (www.sanger.ac.uk/resources/downloads/bacteria/mycobacterium- currently being sequenced and annotated) to determine, *in silico,* if the candidate diagnostics target sequences for *M. bovis* identification were specific. As there was no nucleotide sequence information available for *M. caprae* or *M. pinnipedii* at the time of this study, specificity of the developed assays against these members of the MTC was determined empirically and further validated by nucleotide sequencing.

Since the diagnostics target used in this study for the differentiation of *M. bovis* from the remaining members of the MTC was experimentally determined to also identify *M. caprae* and *M. bovis* BCG, alternative nucleic acid diagnostics targets were required to differentiate this sub-group of the MTC.

For each putative diagnostics target identified, alignments were performed using clustalW multiple sequence alignment programme (http://www.ebi.ac.uk/Tools/clustalw2/index.html), from which real-time PCR primers and probes were designed (Table 1).

**Table 1:** Oligonucleotide primers and probes used in this study.

Name	Purpose	Sequence 5′→3′
MTC_Fw	Forward sequencing primer,forward *M. caprae* real- time PCR assay primer	AGACCGTGCGGATCTTG
MTC_Rv	Reverse sequencing primer, reverse *M. caprae* real-time PCR assay primer	CATGGAGATCACCCGTGA
*M. caprae* specific Probe	*M. caprae* probe	Cyan 500- TATCGGGTACACAAAGACGA-BBQ
lpqT_FW	Forward sequencing primer,forward real-time PCR assay primer	ACGAATCCGGCGATGATC
lpqT_RV	Reverse sequencing primer, reverse real-time PCR assay primer	CGACTGCACACCTGGAA
lpqT Probe	*M. caprae*, *M. bovis*, *M. bovis* BCG probe	FAM-TTGGCCGGCGCCGGTT-BHQ1
RD1_Fw	Forward sequencing primer,forward real-time PCR assay primer	CATCGCTGATGTGCTTGC
RD1_Rv	Reverse sequencing primer, reverse real-time PCR assay primer	TGCGCCGAGCTGTATTC
RD1 Probe	Absence of *M. bovis* BCG	HEX-ACACTAGCGTCAATGCGGTCA-BHQ1
IAC_Fw	Forward sequencing primer,forward real-time PCR assay primer	TCACCGACCATGTCCAG
IAC_RV	Reverse sequencing primer, reverse real-time PCR assay primer	CGTTGCCCAATCCGTATG
IAC probe	IAC probe	CY5-CAGCAGTACCATCGCCATCG-BHQ2

### Bacterial strains, culture media and growth conditions

Seventy two MTC isolates (26 *M. tuberculosis*, 14 *M. bovis*, 7 *M. bovis BCG*, 5 *M. canettii*, 5 *M. caprae*, 5 *M. africanum*, 5 *M. microti* and 5 *M. pinnipedii*), 44 non tuberculosis mycobacteria (NTM) and 17 other bacterial species were used in this study (supplementary Table S1 and Table S2). Of the 72 MTC isolates, 36 strains previously characterised by a variety of methods [24], [25], [26], [27], [28] were provided by The National Institute for Public Health and the Environment (RIVM), the Netherlands. Three *M. caprae*, 3 *M. bovis* and 2 *M. pinnipedii* strains were supplied by the National Reference Centre for Mycobacteria, Borstel, Germany and were previously characterised using a variety of methods [29], [30]. All other MTC strains, provided by Professor Mario Vaneechoutte (University of Ghent, Ghent, Belgium), were clinical isolates collected over a ten year period from reference laboratories in Belgium and the Netherlands. These isolates were characterised on the basis of custom techniques available at the time. Twenty eight NTM were purchased from the German Collection of Microorganisms and Cell Cultures (Deutsche Sammlung von Mikroorganismen und Zellkulturen GmbH, DSMZ) and grown on Middlebook agar/broth at the appropriate temperature (either 30°C or 37°C). Fast growing mycobacteria were cultured for 3-6 days and slow growing mycobacteria were incubated for six weeks, or until sufficient growth was visible. All media were purchased from BD Biosciences (Oxford, United Kingdom). DNA was supplied from the 16 remaining NTM and 4 of the 5 *Norcardia* strains by Professor Vaneechoutte and these species were characterised using techniques previously described [31], [32], [33].

### DNA isolation and quantification

Genomic DNA from 28 NTM and 2 *M. bovis BCG* cultures was isolated from 1 ml of culture (Middlebrook 7H9 broth, Becton Dickinson), using a technique previously described in the literature [23]. Total genomic DNA samples provided by the RIVM were extracted according to Van Soolingen *et al.* [34], whereas total genomic DNA samples from the National Reference Centre for Mycobacteria, Borstel were extracted as described by van Embden *et al.* [35], while genomic DNA provided by Professor Vaneechoutte was extracted as described by De Baere *et al.* [31]. For all other bacterial species tested, DNA was provided from stocks held within this laboratory (Microbiology, NUIG). DNA concentrations for all species and strains used in this study were determined using the PicoGreen dsDNA Quantitation Kit (Molecular Probes, Eugene, Oregon, USA) and the TBS-380 mini-fluorometer (Invitrogen Corporation, California, USA). DNA samples were stored at −20°C before use.

### Conventional and real-time PCR primers and hydrolysis probe design

Oligonucleotide primers and hydrolysis probes were designed in accordance with general recommendations and guidelines [36], [37], following alignments of each of the nucleic acid diagnostics target genes identified in this study. Primers and probes (Table 1) were supplied by MWG-BIOTECH AG (Essenberg, Germany) or TIB MOLBIOL (Berlin, Germany). Primers were designed to have a melting temperature (Tm) of 58–61°C and the probes were designed to have a Tm 4–7°C higher. The probe designed to identify *M. bovis*, *M. bovis* BCG and *M. caprae* had a higher Tm (69.8°C) due to a particularly G/C rich target region. Hydrolysis probes were designed to be specific for each target following published design guidelines [36]. Assay oligonucleotides were designed with similar properties such that monoplex assays could be easily multiplexed after specificity and sensitivity testing was complete [23].

The *M. caprae* specific assay PCR primers, MTC_Fw (position 618–634 bp of the *lepA* gene of *M. tuberculosis* H37Rv) and MTC_Rv (position 754–772 bp), were designed to amplify a 155 bp fragment of the *lepA* gene. lpqT_FW and lpqT_RV were designed to amplify a 141 bp fragment of the *lpqT* gene for the identification of *M. bovis*, *M. bovis* BCG and *M. caprae* (positions 17–34 bp and 146–162 bp of the *M. tuberculosis* H37RV *lpqT* gene). The RD1 assay was designed to amplify a 117 bp region of the Rv3876 gene, a conserved hypothetical protein, part of RD1, absent in all *M. bovis* BCG strains. The RD1_Fw primer was located at position 1416–1433 bp and the reverse primer RD1_Rv between 1516–1532 bp of the *M. tuberculosis* H37 Rv 3876 gene. The internal amplification control (IAC) PCR primers, IAC_Fw and IAC_Rv, were designed to amplify a 157 bp region of the *M. smegmatis* MSMEG_0660 gene. The IAC_Fw primer was located at positions 497–513 bp and the reverse primer between positions 636–653 bp.

All real-time PCR assays were initially tested for in a monoplex format, evaluating their specificity and sensitivity using probes labelled with FAM and Black Hole Quencher 1 (BHQ1). After optimisation of the monoplex real-time PCR assays, three of the four assay probes were labelled with different fluorescent dyes to allow for incorporation into a multiplex real-time PCR format. The *M. caprae* specific probe was labelled with cyan500 and BBQ, the *M. bovis/M. bovis* BCG*/M. caprae* specific probe with FAM and BHQ1, the *M. bovis/M. caprae* probe with HEX and BHQ2 and the IAC probe with Cy5 and BHQ2.

### Conventional PCR

Conventional PCR was performed using the sequencing primers outlined in Table 1 on the iCycler iQ thermal cycler (Bio-Rad Laboratories Inc., California, USA). All reactions were carried out in a final volume of 50 µl, containing 5 µl 10× buffer (15 mM MgCl_2_), 1 µl forward and reverse primers (0.2 µM final conc.), 2 µl Taq DNA polymerase (1 U/µl, Roche Diagnostics, Basel, Switzerland), 1 µl dNTP mix [(10 mM deoxynucleoside triphosphate set (Roche Diagnostics)], 2 µl of template DNA and 38 µl nuclease free water (Applied Biosystems/Ambion, Texas, USA). The cycling parameters consisted of initial denaturation at 95°C for 5 mins, followed by 35 cycles of denaturation at 95°C (1 min), annealing at 50°C (1 min), and extension at 72°C (1 min), with a final elongation step at 72°C for 10 min.

### Sequencing

Nucleotide sequence data for real-time PCR assay design was generated in this study or was obtained from the National Centre for Biotechnology Information (NCBI- http://www.ncbi.nlm.nih.gov/), or the Sanger website (where partial nucleotide sequences for *M. canettii*, *M. africanum* and *M. microti* were available). Real-time PCR assay primers were used to generate nucleotide sequence information for each of the assays developed. Sequencing primers were also designed to span the full gene sequence of *lpqT*, *lepA* and a number of other genes to identify potential candidate nucleic acid diagnostics targets for the specific identification of *M. caprae.*

PCR products were generated according to section 2.5, purified using the High Pure PCR Product Purification Kit (Roche Diagnostics) and sequenced commercially (Sequiserve, Vaterstetten, Germany).

### Development of an IAC for real-time PCR

A non-competitive IAC [38] was designed and incorporated into the multiplex real-time PCR. The *M. smegmatis* MSMEG_0660 gene was chosen as the target as it is specific to *M. smegmatis*, avoiding any possible cross reaction. Titration of IAC DNA was performed to determine the optimum concentration per reaction that could be reliably detected in the presence of MTC DNA, yet not inhibit detection of low concentrations of the primary assay targets. An IAC concentration of 100 genome equivalents per reaction was determined as optimum.

### Real-time PCR

Monoplex real-time PCR was performed on the LightCycler 2.0 Instrument (Roche Diagnostics) using the LightCycler® TaqMan® Master kit (Roche Diagnostics). Each reaction contained 5× master mix, forward and reverse primers (0.5 µM final conc.), FAM labelled probe (0.2 µM final conc.), template DNA (2 µl) and nuclease free dH_2_O to a final volume of 20 µl. The cycling parameters consisted of 10 min incubation at 95°C to activate the Taq, 50 cycles of 95°C for 10 s and 60°C for 30 s, followed by a cooling step at 40°C for 10 s. The temperature transition rate for all cycling steps was 20°C/s.

Multiplex real-time PCR reactions were carried out on the LightCycler 480 using LightCycler® 480 Probes Master kit (Roche Diagnostics). The optimised PCR mix contained 2× LightCycler 480 Probes Master (6.4 mM MgCl_2_), forward and reverse primer (0.5 µM final conc.), Cyan500, FAM, HEX and CY5 labelled probes (0.2 µM final conc.), dimethyl sulfoxide (4%, Sigma-Aldrich Missouri, USA), template DNA (MTC: 2 µl; IAC: 2 µl; NTM: 10 µl) adjusted to a final volume of 40 µl with the addition of nuclease free dH_2_O. The *M. smegmatis* internal control DNA was diluted to contain 100 genome equivalents per 2 µl and NTM DNA was diluted to contain ∼10^4^ genome equivalents per 10 µl.

The cycling parameters used were the same as those used on the LightCycler 2.0. The temperature transition rate, referred to as the ramp rate on the LightCycler 480 was 4.4°C/s while heating and 2.2°C/s while cooling. Prior to experimental analysis on the LightCycler 480, a colour compensation file was generated, to avoid fluorescence leaking from channel to channel, using the technical note outlined in the Advanced Software Functionalities of the operator manual [39].

## Results

### Diagnostics target identification

The diagnostics target used for the specific identification of *M. caprae* was the *lepA* (Rv 2404c) gene. *In-silico* analysis of nucleotide sequences generated in this study revealed a single nucleotide polymorphorism (SNP) at position 690 bp of the *M. tuberculosis* H37Rv *lepA* gene which was specific for *M. caprae*. LepA is an essential bacterial elongation factor. The *lepA* gene codes for a highly conserved protein present in all bacteria sequenced to date, with a homologue (Guf1) found in higher organisms [40]. The *M. caprae* specific SNP, identified from nucleotide sequence data generated in this study, was a C to T substitution which was conserved in all 5 strains of *M. caprae* tested.

The diagnostics target identified in this study for collective identification of *M. bovis*, *M. bovis* BCG and *M. caprae* was the *lpqT* (Rv1016c) gene. LpqT belongs to a group of lipoproteins which are present in all bacteria [41] and is thought to be required for optimal growth of mycobacteria *in vivo* [42]. *In-silico* analysis of publicly available *lpqT* nucleotide sequences, performed in this study, revealed a 5 bp region of *lpqT*, deleted in *M. bovis* and *M. bovis* BCG but present in *M. tuberculosis*, *M. canettii*, *M. africanum* and *M. microti*. Sequence information was generated for the *lpqT* gene in *M. caprae* and *M. pinnipedii* which confirmed the deletion was also present in *M. caprae* but not *M. pinnipedii*.

The diagnostics target used to differentiate *M. bovis* BCG from *M. bovis* and *M. caprae* was the Rv3876 gene, which is part of Region of Difference 1 (RD1). Regions of Difference (RD) in the *Mycobacterium tuberculosis* complex refer to regions of the genome present in *M. tuberculosis* but deleted from *M. bovis* BCG [43]. The particular RD targeted in this study (RD1), has been shown to be deleted in all *M*. *bovis* BCG strains and has been linked to the attenuation of this strain [13]. Any region of RD1 could potentially be incorporated into this multiplex real-time PCR and used with the novel diagnostics targets described above.

### Assay design and development

While the guidelines for primer and probe design were adhered to as closely as possible, the high G/C content (60–65%) of the *Mycobacterium* species had an impact on assay design. The *lpqT* specific probe was designed spanning the deletion junction of a region deleted in *M. bovis, M. bovis* BCG and *M. caprae* and present in the other members of the MTC, that was very G/C rich, making probe design difficult. The *lpqT* probe, therefore, had a relatively high Tm, however this did not impact on assay performance. The *M. caprae* specific probe targeted an SNP in the *lepA* gene. Avoiding cross reaction of the *M. caprae* probe with other members of the MTC proved challenging. A number of probes were designed and tested and the optimum probe was chosen empirically based on specificity and sensitivity results. The optimum probe was designed complementary to the + strand of the *lepA* gene as the resulting G/A mismatch, that occurred in the presence of non-target MTC DNA, was more destabilising to the probe than the C/T mismatch, hence improving specificity. The probe was designed to have a Tm of 60.1°C, only slightly above the annealing temperature of the assay (60°C), allowing the probe to hybridise to exactly matched sequence only, therefore maximising the specificity effect of the SNP. This did, however, slightly reduce probe binding efficiency, leading to a small reduction in sensitivity.

### Internal amplification control

In order for a result to be considered valid using this assay, a positive signal must be obtained in the Cy5 detection channel on the LightCycler 480. If the IAC is not detected, the result is considered invalid and must be repeated [38], [44]. For the purposes of this study, *M. smegmatis* DNA was spiked into the PCR master mix to act as an internal control target. Alternatively, *M. smegmatis* cultured cells could be used as a process control, spiked into patient samples before genomic DNA isolation. In this design, the *M. smegmatis* cells would serve as both an extraction control and an amplification control and could be used when testing patient samples.

### Specificity of the diagnostic assays

**Figure 1:**
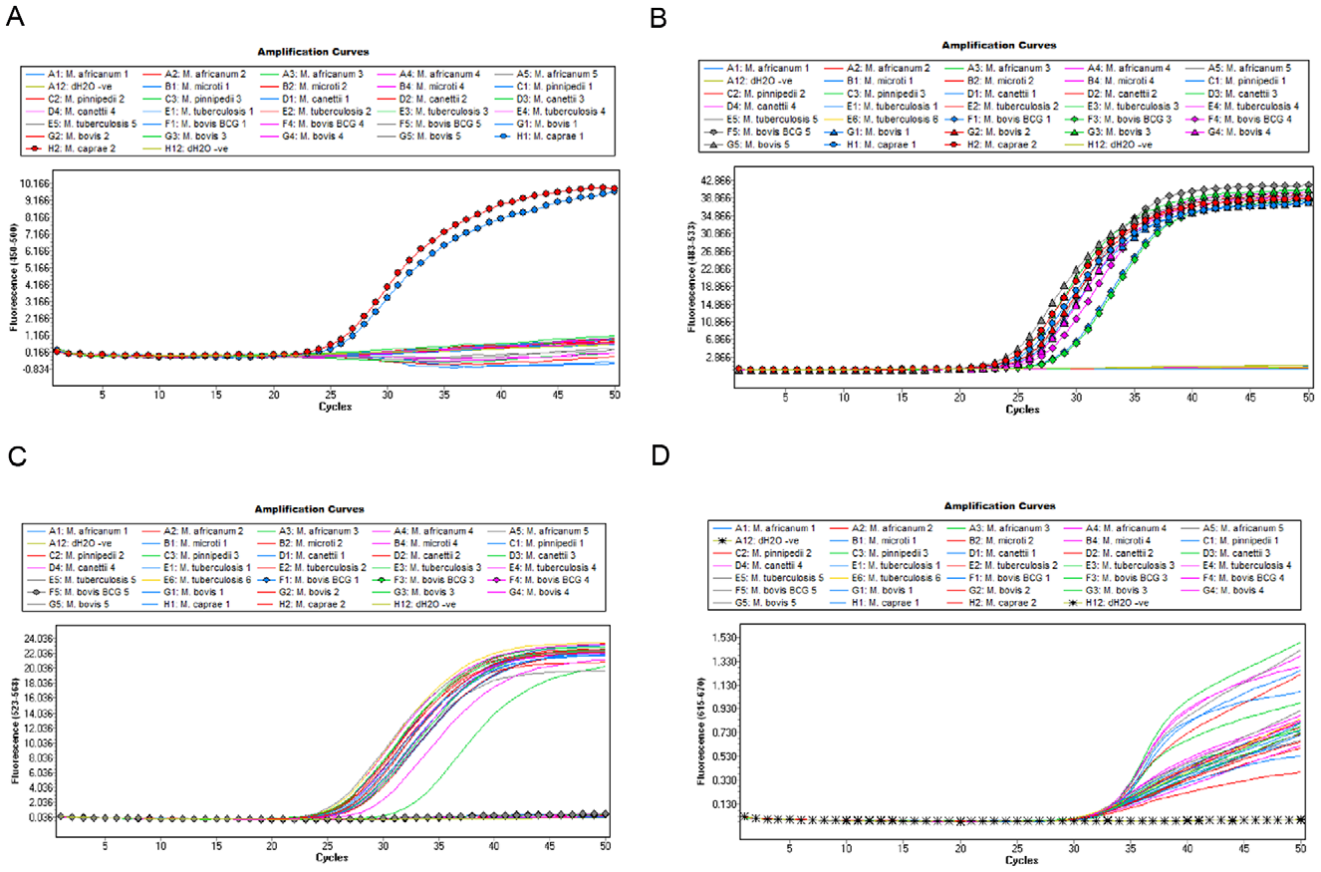
Amplification curves for multiplex real-time PCR assay. Figure 1 A. Real-time amplification curves for *M. caprae* (circle) using *lepA* gene in Cyan500 channel (450–500). Figure 1 B Amplification curves for *M. caprae* (circle), *M. bovis* (triangles) and *M. bovis BCG* (diamonds) using the *lpqT* gene in FAM channel (483–533). Figure 1 C Amplification curves for all members of the MTC with the exception of *M. bovis* BCG (diamonds) and *M. microti* in the HEX channel (523–568). Figure 1 D Amplification curves for IAC in Cy5 channel (615–670) with the no template control highlighted with stars through amplification curve.

The specificity of each real-time PCR assay was confirmed both in monoplex and multiplex formats using the specificity panel listed in supplementary Table S1 and Table S2. The *lepA* assay was specific for the detection of the 5 *M. caprae* strains in our collection (Figure 1 A). The remaining members of the MTC, NTM and other bacteria tested were not detected. The *M. bovis*, *M. bovis BCG* and *M. caprae* assay was also specific, detecting only target species (Figure 1 B). The diagnostics assay targeting RD1 did not detect any of the 7 *M. bovis* BCG strains tested for (Figure 1 C) but detected all *M. bovis* and *M. caprae* strains tested for, allowing for the specific identification of *M. bovis* BCG. All remaining members of the MTC (with the exception of the 5 *M. microti* and one *M. canettii*) were detected. None of the NTM or other bacteria on the specificity panel were detected using the RD1 assay.

The IAC assay targeted the *M. smegmatis* MSMEG_0660 gene. This assay was tested against all members of the MTC and NTM and was specific for the detection of *M. smegmatis*. One hundred genome equivalents of *M. smegmatis* DNA was added to all samples tested, to control for PCR inhibition, and was detected in all samples (Figure 1 D). Table 2 describes how to interpret the results of the multiplex real-time PCR assay.

**Table 2:** Result interpretation table.

Analysis channel result				Diagnostics test result
Cyan 500(+ve indicates presence of *M. caprae*)	FAM(+ve indicates presence of *M*. *bovis*, *M. bovis* BCG or *M. caprae*)	HEX(-ve indicates presence of *M*. *bovis* BCG if positive in FAM channel)	Cy5(IAC)	
+ ve	+ ve	+ ve	+ ve	*M. caprae* present
- ve	+ ve	+ ve	+ ve	*M. bovis* present
- ve	+ ve	- ve	+ ve	*M. bovis BCG* present
- ve	- ve	+ ve	+ ve	MTC member other than *M. caprae, M. bovis*, *M. bovis* BCG or *M. microti*
- ve	- ve	- ve	- ve	Result invalid and test must be repeated

### Sensitivity of the assays

The limit of detection (LOD) of each assay was evaluated in a monoplex real-time PCR format. Genomic DNA was quantified and serial dilutions were prepared from 200,000 to 2 genome equivalents (with an estimated genome size of 4.5 million base pairs, one *M. caprae* cell contains approximately 5.0 fg DNA [45]). Assay sensitivity testing was performed using *M. caprae* DNA as it is detected in all of the multiplex assays.

In a monoplex format, the dilution series was run in duplicate and a sensitivity of 2- 20 *M. caprae* genome equivalents was determined for each assay. In multiplex format the lower limit of detection was established using probit regression analysis. Twelve replicates of each of 20, 15, 12, 10, 7.5, 4, 2 and 0.2 *M. caprae* genome equivalents were tested. LOD's of 3.95 and 3.55 genome equivalents were determined (95% probability) for the *M. bovis*/*M. bovis* BCG/*M. caprae* and the *M. bovis*/*M. caprae* assays respectively. However, these dilutions were not appropriate to determine the *M. caprae* specific assay LOD. The analysis was repeated using concentrations of 200, 100, 75, 50, 25, 15, 10 and 5 genome equivalents of *M. caprae* DNA. An LOD of 14.77 genome equivalents (95% probability) was determined for the *M. caprae* specific assay when tested as part of the multiplex. The IAC, at a concentration of 100 genome equivalents per reaction, was included in all samples during sensitivity testing and was detected as expected.

## Discussion

The WHO [46] defines zoonoses as infections and diseases which can be transmitted naturally from animals to humans. A recent WHO report highlights the difficulties in the diagnosis of such diseases and suggests that the true incidence of many neglected zoonoses, including zoonotic TB, may be greatly underestimated [46]. Zoonotic TB in humans is most commonly caused by *M. bovis* and to a lesser extent *M. caprae* (prevalent in certain regions in Europe [47]). Human TB infection caused by the zoonotic transmission of *M. microti* and *M. pinnipedii* has also been reported [48], [49], [50]. While bovine TB in humans is considered rare in developed countries, it has been described as an ‘endemic neglected zoonoses’ in developing countries [51]. In these settings, the true incidence of zoonotic TB in humans remains largely unknown due to inabilities in hospital laboratories to distinguish between the causative agent of infection, a scarcity of trained personnel, financial constraints in addition to limited control measures in place [51], [52].

*M. bovis* is the predominant member of the MTC associated with bovine TB infection. However, it also has been isolated from and is the causative agent of TB in a wide range of mammalian hosts including humans, goats, dogs, deer and ferrets [10]. Transmission to and infection of human hosts with *M. bovis* can result from zoonotic exposure to TB infected animals or to consumption of unpasteurised dairy products [8]. Recent studies have also shown that human to human transmission of *M. bovis* can also occur, but is considered rare [53].

Since its transfer from *M. tuberculosis* subsp *caprae* to *M. bovis* subsp *caprae* in 2002 [5], this member of the MTC has been recognised as the cause of a number of outbreaks of TB in animals such as goats, cattle and red deer [47]. In addition, *M. caprae* has been associated with outbreaks of TB in humans in a number of European countries [54], [55], [56] and was shown to be the true causative agent of one third of human *M. bovis* associated TB cases in Germany between 1999–2001 [10].

*M. bovis* BCG, the commonly used vaccine strain, has also been implicated in human disease particularly in immunocompromised patients, such as HIV positive and cancer patients, where it has been shown to cause disseminated disease [57], and in children who have been vaccinated in endemic TB areas [58].

While the members of the MTC discussed above share many phenotypic characteristics, in addition to natural host preference, *M. bovis, M. bovis* BCG and *M. caprae* exhibit a differential intrinsic susceptibility profile to pyrazinamide (PZA), an important first line anti-TB drug responsible for reducing patient treatment time [59]. *M. bovis* and *M. bovis* BCG are intrinsically resistant to PZA whereas *M. caprae* is sensitive to PZA [5]. This highlights the need for a rapid test that can detect the MTC and differentiate between its members so that the clinician can choose the appropriate chemotherapy for the patient. More accurate diagnosis and treatment will protect against the development of drug resistant TB and reduce morbidity and mortality [10]. Furthermore, accurate identification of these organisms allows monitoring of these neglected members of the MTC with respect to prevalence, transmission and global distribution [48]. Also, differentiation of the complex could contribute to the better understanding of the clinical relevance of these members of the MTC.

Currently, there is only one commercially available diagnostics kit for the differentiation of the MTC, the GenoType MTBC line probe assay (Hain Lifesciences). This assay differentiates members of the MTC utilising highly conserved SNP's in the *gyrB* gene [60], however it is limited as it does not differentiate *M. canettii* and *M. pinnipedii*. Literature describing NAAT's for the complete differentiation of the MTC [50], [61], [62], [63] are hindered by the complexity of the multiple reactions required, the inability to differentiate between some members of the MTC, in addition to the complex interpretation of the results generated. While SNP's have commonly been used for accurate differentiation of members of the MTC [3], [50] further validation of the SNP identified in this study for the specific detection of *M. caprae* is required.

The multiplex real-time PCR assay described in this study is the first description of a real-time PCR diagnostics assay for the identification and simultaneous differentiation of *M. bovis*, *M. bovis* BCG and *M. caprae* using novel diagnostics targets in one internally controlled reaction. This diagnostics assay takes approximately one hour to perform after DNA extraction. The assay has been validated against a large panel of well characterised clinical isolates. In its current format, this multiplex real-time PCR assay would be applied to MTC clinical isolates for the identification of the most common causes of zoonotic TB. Monitoring bovine TB in humans is valuable for guiding public health policy and is important for the study of zoonotic TB epidemiology.

The developed assay also has the potential to be used directly in sputum samples following identification of an efficient sputum sample preparation method. If performing direct sputum testing, the assay developed in this study would be used as a follow on test in combination with a real-time PCR assay for identification of the MTC and differentiation of *M. tuberculosis* and *M. canettii,* which was previously developed by this group. More specifically, if a patient sample is identified as positive for the presence of the MTC but *M. tuberculosis* or *M. canettii* negative, the assay developed in this study can then be used to determine if sample contains *M. bovis, M. bovis* BCG or *M. caprae*, the most common causes of zoonotic TB in humans.

The study presented here focuses on accurately differentiating members of the MTC most commonly associated with zoonotic human TB infection. However, there is a need to differentiate between all members of the MTC to supply unambiguous epidemiological data as to the true prevalence of each member of the MTC causing TB infection [3]. Work has begun in this group on the development of a final multiplex real-time PCR diagnostics assay for the accurate identification of *M. africanum*, *M. microti* and *M. pinnipedii*. This will complete a cassette of three multiplex real-time PCR diagnostics assays for the identification and differentiation of all members of the MTC.

## Supporting Information

Table S1 Description of *Mycobacterium tuberculosis* complex isolates used in this study.
(DOC)

Table S2 Description of other Non *tuberculosis* mycobacteria and other strains of bacteria used in this study.
(DOC)
