# Supplementary material for: A Novel Multiplex Real-Time PCR for the Identification of Mycobacteria Associated with Zoonotic Tuberculosis
Source: PLoS One. 2011 Aug 9;6(8):e23481. doi: 10.1371/journal.pone.0023481 (PMC3153498; doi:10.1371/journal.pone.0023481)
Supplement: Table S1 — Description of Mycobacterium tuberculosis complex isolates used in this study. (DOC) [file pone.0023481.s001.doc]

Table S1: *Mycobacterium tuberculosis* complex isolates used in this study

| Species | Strain/ collection number ^a^ | Country of Isolation | DNA provided by |
| --- | --- | --- | --- |
| *M. tuberculosis* | 22 | Mongolia | RIVM, Bilthoven, the Netherlands |
| *M. tuberculosis* | 53 | Argentina | RIVM, Bilthoven, the Netherlands |
| *M. tuberculosis* | 112 | The Netherlands | RIVM, Bilthoven, the Netherlands |
| *M. tuberculosis* | 67 | Comoro Islands | RIVM, Bilthoven, the Netherlands |
| *M. tuberculosis* | 41 | Chile | RIVM, Bilthoven, the Netherlands |
| *M. tuberculosis* | 103 | China | RIVM, Bilthoven, the Netherlands |
| *M. tuberculosis* (*19 clinical isolates*) | - | NA^c^ | Mario Vaneechoutte, University of Ghent, Ghent, Belgium |
| *M. tuberculosis* H37Rv | H37Rv | NA^c^ | DSMZ |
| *M. canettii* | 116 | Somalia | RIVM, Bilthoven, the Netherlands |
| *M. canettii* | 1997-1549 | Switzerland | RIVM, Bilthoven, the Netherlands |
| *M. canettii* | NLA000701671 | Somalia | RIVM, Bilthoven, the Netherlands |
| *M. canettii* | NLA000200937 | Eritrea | RIVM, Bilthoven, the Netherlands |
| *M. canettii* | 1996-46 | France | RIVM, Bilthoven, the Netherlands |
| *M. bovis* | 117 | Argentina | RIVM, Bilthoven, the Netherlands |
| *M. bovis* | 126 | Argentina | RIVM, Bilthoven, the Netherlands |
| *M. bovis* | 73 | The Netherlands | RIVM, Bilthoven, the Netherlands |
| *M. bovis* | 130 | The Netherlands | RIVM, Bilthoven, the Netherlands |
| *M. bovis* | 24 | Saudi Arabia | RIVM, Bilthoven, the Netherlands |
| *M. bovis* | 4258_00 | Germany | Research Center Borstel, Germany |
| *M. bovis* | 751_01 | Germany | Research Center Borstel, Germany |
| *M. bovis* | 7540_01 | Germany | Research Center Borstel, Germany |
| *M. bovis*  (6 isolates) | - | NA^c^ | Mario Vaneechoutte, University of Ghent, Ghent, Belgium |
| *M. bovis* BCG | 48 (2) | The Netherlands | RIVM, Bilthoven, the Netherlands |
| *M. bovis* BCG | 71 | Japan | RIVM, Bilthoven, the Netherlands |
| *M. bovis* BCG | 83 | Russia | RIVM, Bilthoven, the Netherlands |
| *M. bovis* BCG | 2008-714 ^b^ | NA^c^ | RIVM, Bilthoven, the Netherlands |
| *M. bovis* BCG | 2008-1601 ^b^ | NA^c^ | RIVM, Bilthoven, the Netherlands |
| *M. bovis* BCG | DSM 43990 | NA^c^ | DSMZ |
| *M. bovis* BCG | DSM 45071 | NA^c^ | DSMZ |
| *M. caprae* | 2006-1960 ^b^ | The Netherlands | RIVM, Bilthoven, the Netherlands |
| *M. caprae* | 2007-0039 ^b^ | The Netherlands | RIVM, Bilthoven, the Netherlands |
| *M. caprae* | 1694_00 | Germany | Research Center Borstel, Germany |
| *M. caprae* | 8986_99 | Germany | Research Center Borstel, Germany |
| *M. caprae* | 9577_99 | Germany | Research Center Borstel, Germany |
| *M. microti* | 62 | United Kingdom | RIVM, Bilthoven, the Netherlands |
| *M. microti* | 25 | United Kingdom | RIVM, Bilthoven, the Netherlands |
| *M. microti* | 15274 ^b^ | United Kingdom | RIVM, Bilthoven, the Netherlands |
| *M. microti* | 15912 ^b^ | Belgium | RIVM, Bilthoven, the Netherlands |
| *M. microti* | 15911 ^b^ | Netherlands | RIVM, Bilthoven, the Netherlands |
| *M. pinnipedii* | 76 | Argentina | RIVM, Bilthoven, the Netherlands |
| *M. pinnipedii* | 81 | Argentina | RIVM, Bilthoven, the Netherlands |
| *M. pinnipedii* | 101 | Argentina | RIVM, Bilthoven, the Netherlands |
| *M. pinnipedii* | 7011_02 | Germany | Research Center Borstel, Germany |
| *M. pinnipedii* | 7739_01 | Germany | Research Center Borstel, Germany |
| *M. africanum* | 6 | The Netherlands | RIVM, Bilthoven, the Netherlands |
| *M. africanum* | 128 (85) | The Netherlands | RIVM, Bilthoven, the Netherlands |
| *M. africanum* | 2007-1386 ^b^ | The Netherlands | RIVM, Bilthoven, the Netherlands |
| *M. africanum* | 2007-1154 ^b^ | The Netherlands | RIVM, Bilthoven, the Netherlands |
| *M. africanum* | 2007-1073 ^b^ | The Netherlands | RIVM, Bilthoven, the Netherlands |

^a^ RIVM = National Tuberculosis Reference Laboratory, National Institute for Public Health and the Environment, Bilthoven, The Netherlands; *DSM = The German Collection of Microorganisms; *Research Center Borstel = Germany National Reference Center for Mycobacteria, Forschungszentrum Borstel, Germany

^b^ Represent RIVM strains not previously described in literature, however have been characterised to the species level using techniques outlined in references supplied in the manuscript.

^c^ This information was not available (NA) for this study.
