# Supplementary material for: A Novel Multiplex Real-Time PCR for the Identification of Mycobacteria Associated with Zoonotic Tuberculosis
Source: PLoS One. 2011 Aug 9;6(8):e23481. doi: 10.1371/journal.pone.0023481 (PMC3153498; doi:10.1371/journal.pone.0023481)
Supplement: Table S2 — Description of other Non tuberculosis mycobacteria and other strains of bacteria used in this study. (DOC) [file pone.0023481.s002.doc]

Table S2: Non *tuberculosis* mycobacteria and other strains of bacteria used in this study

| **Non *tuberculosis*  mycobacteria** | **Strain designation** ^a^ | **Remark** |
| --- | --- | --- |
| *Mycobacterium aichiense* | DSM 44147 | Type strain, isolated from soil |
| *Mycobacterium alvei* | DSM 44176 | Type strain, isolated from water sample |
| *Mycobacterium arupense* | DSM 44942 | Type strain, isolated from a tendon |
| *Mycobacterium asiaticum* | ITG 8182 | See De Baere *et al.* 2002 |
| *Mycobacterium avium* | ITG 7886 | See Vaneechoutte *et al.* 1993 |
| *Mycobacterium boenickei* | DSM 44677 | Type strain, isolated from a leg wound |
| *Mycobacterium branderi* | DSM 44624 | Type strain, isolated from human sputum |
| *Mycobacterium brisbanense* | DSM 44680 | Type strain, isolated from a sinus |
| *Mycobacterium brumae* | DSM 44177 | Type strain, isolated from water sample |
| *Mycobacterium canariasense* | DSM 44828 | Type strain, isolated from human blood |
| *Mycobacterium celatum* | ITG 6147 | See De Baere *et al.* 2002 |
| *Mycobacterium chelonae* | ITG 4975 | NA ^b^ |
| *Mycobacterium chelonae subsp. abscessus* | DSM 44196 | Type strain |
| *Mycobacterium confluentis* | DSM 44017 | Type strain, isolated from human sputum |
| *Mycobacterium conspicuum* | DSM 44136 | Type strain, isolated from patient with disseminated infection |
| *Mycobacterium flavescens* | VUB A016 | See De Baere *et al.* 2002 |
| *Mycobacterium fortuitum* | ITG 8020 | See Vaneechoutte *et al.* 1993 |
| *Mycobacterium genavense* | ITG 97-102 | See De Baere *et al.* 2002 |
| *Mycobacterium gilvum* | DSM 9487 | Isolated from soil |
| *Mycobacterium goodii* | DSM 44492 | Type strain |
| *Mycobacterium gordonae* | ITG 7704 | See Vaneechoutte *et al.* 1993 |
| *Mycobacterium heckeshornense* | DSM 44428 | Type strain, isolated from human respiratory tract |
| *Mycobacterium houstonense* | DSM 44676 | Type strain, isolated from a facial abscess |
| *Mycobacterium intracellulare* | DSM 43223 | Type strain |
| *Mycobacterium kansasii* | ITG 7727 | See Vaneechoutte *et al.* 1993 |
| *Mycobacterium kubiciae* | DSM 44627 | Type strain, isolated from human sputum |
| *Mycobacterium lacus* | DSM 44577 | Type strain, isolated from human elbow |
| *Mycobacterium mageritense* | DSM 44476 | Type strain, isolated from human sputum |
| *Mycobacterium malmoense* | ITG 940611 | See De Baere *et al.* 2002 |
| *Mycobacterium marinum* | ITG 1727 | See Vaneechoutte *et al.* 1993 |
| *Mycobacterium massiliense* | DSM 45103 | Type strain, isolated from human blood |
| *Mycobacterium moriokaense* | DSM 44221 | Type strain, isolated from soil |
| *Mycobacterium mucogenicum* | DSM 44625 | Type strain, isolated from human cyst |
| *Mycobacterium nebraskense* | DSM 44803 | Type strain, isolated from human sputum |
| *Mycobacterium neworleansense* | DSM 44679 | Type strain, isolated from human scalp |
| *Mycobacterium paratuberculosis* | ITG 2666 | See De Baere *et al.* 2002 |
| *Mycobacterium scrofulaceum* | DSM 43992 | Type strain, isolated from human cervical lymph node |
| *Mycobacterium shimoidei* | DSM 44152 | Type strain, isolated from sputum of patient with tuberculosis-like disease |
| *Mycobacterium simiae* | ITG 4485 | See Vaneechoutte *et al.* 1993 |
| *Mycobacterium smegmatis* | DSM 43756 | Type strain |
| *Mycobacterium szulgai* | ITG 4979 | NA ^b^ |
| *Mycobacterium tusciae* | DSM 44338 | Type strain, isolated from human cervical lymph node |
| *Mycobacterium ulcerans* | ITG 96-1439 | NA ^b^ |
| *Mycobacterium xenopi* | ITG 4986 | See De Baere *et al.* 2002 |
| **Other bacteria** | **Strain designation** | **Remark** |
| *Staphylococcus aureus* | DSM 20231 | Type strain, isolated from human pleural fluid |
| *Listeria monocytogenes* | DSM 20600 | Type strain, isolated from a rabbit |
| *Escherichia coli* | DSM 301 | Disinfectant test strain |
| *Klebsiella oxytoca* | ATCC 43086 |  |
| *Enterococcus faecalis* | DSM 20371 | Isolated from pleural fluid |
| *Proteus mirabilis* | DSM 4479 | Type strain |
| *Bacillus cereus* | DSM 31 | Type strain |
| *Bordetella pertussis* | CCUG 13475 | Isolated from patient suffering from whooping cough |
| *Streptococcus agalactiae* | DSM 2134 | Type strain |
| *Rhodococcus equi* | DSM 20307 | Type strain, isolated from lung abscess of foal |
| *Streptomyces albidoflavus* | DSM 40455 | Type strain |
| *Nocardioides sp.* | DSM 17401 | Proposed type strain, isolated from marine sediment |
| ***Nocardia salmonicida*** | DSM 40472 | Type strain, isolated from blueback salmon |
| *Nocardia asiatica* | clinical isolate | Isolated from human wound, see Wauters *et al.* 2005 |
| *Nocardia nova* | clinical isolate | Isolated from human abscess, see Wauters *et al.* 2005 |
| *Nocardia cyriacigeorgica* | clinical isolate | Isolated from human bronchial aspirate, see Wauters *et al.* 2005 |
| *Nocardia farcinica* | clinical isolate | Isolated from human abscess, see Wauters *et al.* 2005 |

^a^ DSM = The German Collection of Microorganisms; *ATCC = American Type Culture Collection; *ITG = Institute of Tropical Medicine, Antwerp, Germany; *CCUG = Culture Collection, University of Göteborg, Sweden; *VUB = Department of Microbiology, Academic Hospital of the Free University of Brussels, Brussels, Belgium.

^b^ This information was not available (NA) for this study.
